# Liver Injury Induced by Carbon Tetrachloride in Mice Is Prevented by the Antioxidant Capacity of Anji White Tea Polyphenols

**DOI:** 10.3390/antiox8030064

**Published:** 2019-03-14

**Authors:** Ranran Wang, Zhiqing Yang, Jing Zhang, Jianfei Mu, Xianrong Zhou, Xin Zhao

**Affiliations:** 1 Chongqing Collaborative Innovation Center for Functional Food, Chongqing University of Education, Chongqing 400067, China; wangranran@foods.ac.cn (R.W.); mujianfei@foods.ac.cn (J.M.); zhouxr@foods.ac.cn (X.Z.); 2 Chongqing Engineering Research Center of Functional Food, Chongqing University of Education, Chongqing 400067, China; 3 Chongqing Engineering Laboratory for Research and Development of Functional Food, Chongqing University of Education, Chongqing 400067, China; 4 College of Biological and Chemical Engineering, Chongqing University of Education, Chongqing 400067, China; yangzq@foods.ac.cn; 5 Environment and Quality Inspection College, Chongqing Chemical Industry Vocational College, Chongqing 401228, China; zhangjing@foods.ac.cn; 6 College of Food Science, Southwest University, Chongqing 400715, China

**Keywords:** Anji white tea, polyphenol, liver injury, mice, mRNA expression

## Abstract

Anji white tea is a unique variety of green tea that is rich in polyphenols. In this study, the effect of Anji white tea polyphenols (AJWTP) on the prevention of carbon tetrachloride (CCl_4_)-induced liver injury through its antioxidant properties was studied. Biochemical and molecular biology methods were used to analyze the serum and liver tissue of mice. The antioxidant capacity and liver injury preventive effect of AJWTP were determined, and the mechanism was elaborated. The results showed that AJWTP decreased the serum levels of aspartate aminotransferase (AST), alanine aminotransferase (ALT), triglyceride (TG), and total cholesterol (TC) in mice with liver injury, it increased the activities of superoxide dismutase (SOD) and glutathione peroxidase (GSH-Px) in the serum and liver tissue of mice with liver injury, and it also decreased the amount of malondialdehyde (MDA). Further quantitative polymerase chain reaction (qPCR) results showed that AJWTP upregulated the mRNA expression of *Cu/Zn-SOD*, *Mn-SOD*, catalase (*CAT*), and nuclear factor of kappa light polypeptide gene enhancer in B-cell inhibitor alpha (*IκB-α*) and downregulated the expression of nuclear factor κ-light-chain-enhancer of activated B-cells (*NF-κB*), cyclooxygenase-2 (*COX-2*), inducible nitric oxide synthase (*iNOS*), interleukin-1 beta (*IL-1β*), and tumor necrosis factor alpha (*TNF-α*) in the liver tissue of mice with liver injury. Therefore, AJWTP produces sufficient antioxidant action to prevent liver injury, and the effect increases with the increase in AJWTP concentration. The effect of 200 mg/kg AJWTP was similar to that of the same concentration of the drug (silymarin) used for the treatment of liver injury. This indicates excellent potential for the development and utilization of AJWTP because it is an active substance with excellent antioxidant effects and can prevent liver injury.

## 1. Introduction

The liver is the largest metabolic organ in the body, and plays an important role in the metabolic activities of the human body. Most of the substances consumed or absorbed by the body are transformed through the metabolism of the liver [1]. Acute liver failure (ALF) is a difficult research topic because of its acute onset, rapid progress, many complications, difficult treatment, high mortality, and poor prognosis. Carbon tetrachloride (CCl_4_) is a typical hepatotoxic substance, and its mechanism of action is complex. Study has shown that CCl_4_ is related to oxidative stress and lipid peroxidation. When CCl_4_ enters hepatocytes, it is activated by cytochrome P450 enzyme metabolism to produce trichloromethyl free radicals (CCl_3_•) and trichloromethyl peroxide free radicals (OOCCl_3_•). These free radicals covalently bind with phospholipid molecules on hepatocyte membranes, endoplasmic reticulum, and mitochondria, which induces lipid peroxidation to subsequently damage the structure and function of membranes, and inhibit cell membranes and mitochondria [2,3]. The activity of calcium pumps on granulosa membranes causes a large amount of Ca^2+^ influx, which leads to hepatocyte damage, thus affecting the normal material and energy metabolism of cells [4,5]. Byproducts of lipid peroxidation, such as reactive aldehydes, can also bind to normal intracellular proteins and DNA and result in hepatotoxicity and carcinogenicity [6].

The structural characteristics of plant polyphenols provide them with strong antioxidant and free radical scavenging abilities. The phenolic hydroxyl structure, especially the ortho-phenolic hydroxyl in catechol or pyrogallol, can easily be oxidized to a quinone structure. It has a strong ability to capture free radicals, such as reactive oxygen species, or lipid free radicals produced by oxidation reactions, to reduce or prevent the oxidation reaction in tissues [7]. In vivo studies showed that plant polyphenols have many biological activities through their free radical scavenging effects [8,9]. Plant polyphenols can effectively increase the vitality of the important antioxidant enzymes, superoxide dismutase (SOD), glutathione peroxidase (GSH-Px), and catalase (*CAT*), in the body and play an antioxidant role [10]. Tea polyphenols perform biological activities, such as scavenging free radicals and antioxidants [11]. For experimental liver injury induced by CCl_4_, lipid peroxidation induced by CCl_4_, and covalent binding of CCl_4_ with liver microsome lipids and proteins can be effectively reduced by tea polyphenols [12].

Anji white tea (AJWT) is mainly produced in Anji County, Zhejiang Province, China, and is named for its place of origin. The raw material of AJWT is the leaves of a tea plant, which are light green because of a chlorophyll deficiency caused by variance, and its tea soup is almost colorless. Tea products are made according to green tea processing technology and are classified as non-fermented tea [13]. AJWT is rich in polyphenols. In vitro study has confirmed that Anji white tea polyphenols (AJWTP) have a scavenging effect on superoxide anions (O_2_^−^•) and hydroxyl radicals (OH), an ability to form a complex with Fe^2+^, and can inhibit erythrocyte hemolysis and erythrocyte hemolysis induced by hydrogen peroxide (H_2_O^2^) [14]. In addition, AJWTP can also significantly decrease the experimental oxidative damage caused by D-galactose [15]. In vitro and in vivo experiments have preliminarily proven that AJWTP has antioxidant effects, but there is no corresponding research on the prevention or treatment of other diseases through its antioxidant capacity.

Carbon tetrachloride produces free radicals, CCl_3_, catalyzed by cytochrome P450. Free radicals attack the biofilm and cause lipid peroxidation, which results in acute liver injury. In this study, CCl_4_ was used to establish an oxidative liver injury model in mice. Silymarin is a recognized drug for the treatment of liver injury. In this study, it was used as a positive control. At the same time, the dosage of mice (200 mg/kg body weight (b.w.)) was converted according to the dosage for the human body. The ability of AJWTP extract to ameliorate CCl_4_-induced liver injury in mice was then observed. Molecular biological methods were used to detect the related indexes of mouse serum and liver tissues, and the mechanism of the improvement of liver injury in mice by AJWTP through its antioxidant capacity was explained. The research results will benefit the development and utilization of AJWTP extract, and provide a theoretical basis for the development of AJWTP in the research and development of food processing and health products.

## 2. Materials and Methods

### 2.1. AJWTP Extraction

**Figure 1:**
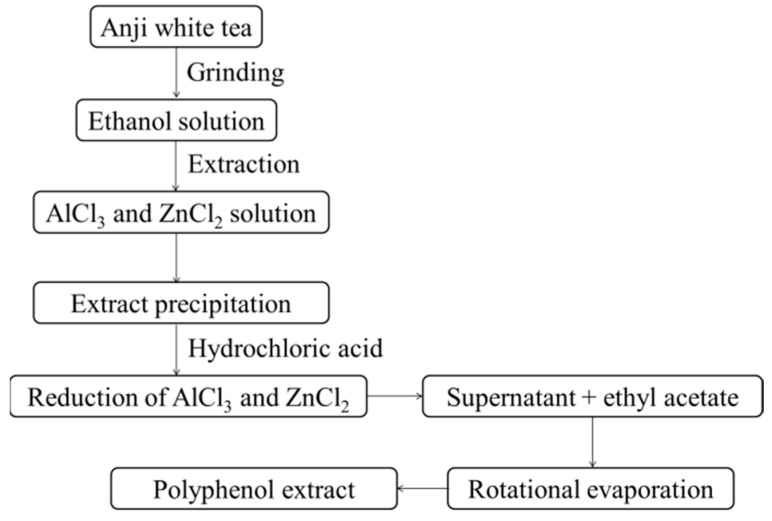
Polyphenol extraction process of this study.

One-hundred grams of Anji white tea (AJWT) was weighed and crushed to a powder, 45% (volume ratio) of 150 mL of ethanol solution was added, and extraction was performed at 90 °C for 30 min (Figure 1). After repeated extraction, the pH of the extract was adjusted to 6.0, 6 g of AlCl_3_ and 12 g of ZnCl_2_ were dissolved in 160 mL of deionized water and then added to the extract for precipitation, and the mixture was centrifuged at 3000 rpm for 10 min. Next, 200 mL of 12% hydrochloric acid (volume ratio) was added to the collected precipitation for transsolution, the supernatant was separated, and 200 mL of ethyl acetate was added twice for extraction. Finally, the liquid was evaporated to obtain the polyphenol extract [16]. 

### 2.2. Determination of Anji White Tea Polyphenol (AJWTP) Content

Chlorogenic acid was dissolved in distilled water, which was then diluted to obtain different concentrations of chlorogenic acid solution. According to the Folin-Ciocalteu colorimetric method, 1 mL of chlorogenic acid solution at different concentrations, 3 mL of Folin-Ciocalteu colorimetric agent, and 4.5 mL of saturated Na_2_CO_3_ solution were mixed and brought to a volume of 25 mL. The absorbance value of the decolorized solution was measured at 747 nm. The standard concentration of chlorogenic acid was plotted with the absorbance value as the *x*-axis coordinate, and the concentration of chlorogenic acid as the *y*-axis coordinate. AJWTP was dissolved in distilled water, and the content of AJWTP was determined using the standard curve by the above method.

### 2.3. Animal Experiments

**Figure 2:**
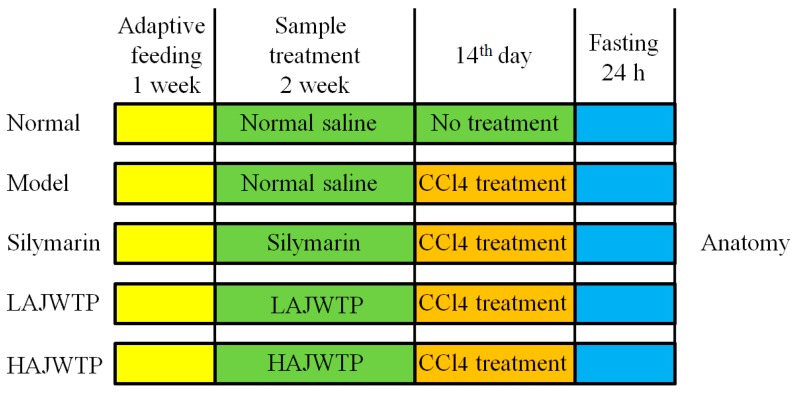
Animal experiment design of this study.

Fifty specific pathogen free (SPF) 6-week-old male Kunming (KM) mice were fed for one week and then were divided into five groups: The normal group, model group, silymarin group, low concentration AJWTP (LAJWTP) group, and high concentration AJWTP (HAJWTP) group, with 10 mice in each group. For 14 days, mice in the normal group and model group were given normal saline by gavage, mice in the silymarin group were given silymarin at a concentration of 200 mg/kg, and mice in the LAJWTP group and HAJWTP group were given AJWTP at a concentration of 100 and 200 mg/kg by gavage, respectively. On the 14th day, mice in the model group, silymarin group, and AJWTP groups were intraperitoneally injected with CCl_4_ solution (0.8% CCl_4_ solution) at a dose of 0.1 mL/10 g. After intraperitoneal injection of CCl_4_ solution, all experimental mice were fasted for 24 h (Figure 2). Then, the mice were euthanized, and the liver and blood was removed for analysis. The liver index is an index to measure liver injury in animal experiments. Additionally, the liver index was determined using the formula: Liver index (%) = organ weight (g)/body weight of mice (kg) × 100 [17]. This study was conducted in accordance with the Declaration of Helsinki, and the protocol was approved by the Ethics Committee of Chongqing Collaborative Innovation Center for Functional Food (201803002B).

### 2.4. Pathological Observation of Liver Tissue in Mice

The liver tissue of mice was immobilized in 10% formalin solution for 48 h. The liver tissue was dehydrated, made transparent, waxed, embedded, and sectioned. Then, the liver tissue was stained with hematoxylin and eosin (H&E), and the morphological changes were observed under an optical microscope (BX43, Olympus, Tokyo, Japan).

### 2.5. Determination of Aspartate Aminotransferase (AST), Alanine Aminotransferase (ALT), Triglyceride (TG), and Total Cholesterol (TC) Serum Levels in Mice

The whole blood of the mice was maintained for 1 h at room temperature. Then, the blood was centrifuged at 4000 rpm for 10 min, and the upper serum was removed. The levels of AST, ALT, TG, and TC in the serum of the mice were determined according to kits (Nanjing Jiancheng Bioengineering Institute, Nanjing, China). 

### 2.6. Determination of Malondialdehyde (MDA) Content and SOD and GSH-Px Activities in the Serum and Liver Tissues in Mice

Mouse liver tissue was homogenized at 10% and centrifuged at 4000 rpm for 10 min. The supernatant was removed for determination. The MDA content and SOD and GSH-Px activities in liver tissues were determined according to kits (Nanjing Jiancheng Bioengineering Institute, Nanjing, China).

### 2.7. Quantitative PCR (qPCR) Assay

The liver tissue of mice was crushed and the total RNA was extracted by RNAzol (Thermo Fisher Scientific, Inc., Waltham, MA, USA) and diluted to 1 µg/µL. The diluted total RNA solution of 5 µL was reverse transcribed with a kit (Quantscript RT kit, Tiangen Biotech Co., Ltd., Beijing, China), and the DNA template was obtained. For the qPCR, 2 µL of DNA template was mixed with 10 µL of SYBR Green PCR Master Mix (Thermo Fisher Scientific, Inc., Waltham, MA, USA) and 1 µL of (100 nmol/mL) forward and reverse primers (Table 1), reacted at 95 °C for 60 s, then 40 cycles were performed at 95 °C for 15 s, 55 °C for 30 s, and 72 °C for 35 s. Finally, the gene expression was detected at 95 °C for 30 s, and 55 °C for 35 s by a real time PCR instrument (SteponePlus, Thermo Fisher Scientific, Inc., Waltham, MA, USA). *GAPDH* was used as an internal reference, and the relative gene expression was calculated using the 2^−ΔΔCt^ method [18].

### 2.8. Western Blot

Protein was extracted from liver tissue by 1 mL of radio-immunoprecipitation assay (RIPA) and 10 L of phenylmethylsulfonyl fluoride (PMSF) (Thermo Fisher Scientific, Inc., Waltham, MA, USA). A bicinchoninic acid (BCA) protein quantitative kit was used to quantify protein. The sample proteins were diluted to 50 g/mL. The diluted proteins were mixed with sample buffer at 4:1 and then heated for 5 min at 100 °C. Mixing Acrylamide, Resolving Buffer, Starcking Buffer, Ditilled Water, 10% APS, and TEMED were proportionally mixed to form SDS-PAGE (25 °C) separating and concentrating glue, which was poured into the runner board for later use. Prestained Protein Ladder and samples were put into the sample hole of the rubber sheet, respectively, and then the SDS-PAGE glue containing protein was subjected to 50 min vertical gel electrophoresis. The PVDF membrane (Thermo Fisher Scientific, Inc., Waltham, MA, USA) was activated by methanol for 1 min and then the transmembrane was closed by TBST solution containing 5% skim milk for 1 h. After closure, the PVDF membrane was washed by TBST, the first resistance was incubated at 25 °C for 2 h, and the second resistance was incubated at 25 °C for 1 h. Finally, Supersignal West Pico PLUS was used to spray the PVDF film and was placed in iBright FL1000 for observation (Thermo Fisher Scientific, Inc., Waltham, MA, USA) [17]. 

### 2.9. Statistical Analysis

The serum and tissue tests for each mouse were performed three times in parallel, and then the average was taken. SAS 9.1 statistical software (SAS Institute Inc., Cary, NC, USA) was used to analyze the data. The one-way ANOVA method according to Duncan’s multiple-range test was used to analyze whether there were significant differences between any two groups of data at the level of *p* < 0.05 [18].

## 3. Results

### 3.1. Content of AJWTP

The regression equation of the standard curve of the chlorogenic acid standard solution is:

*Y* = 0.005*X* + 0.02415 (*R*^2^ = 0.9964) (1)

where *Y* denotes the absorbance value, and *X* denotes the chlorogenic acid concentration. According to the standard curve calculation, the content of extracted AJWTP (chlorogenic acid meter) is 80.9%, which proves that the main component of the experimental sample is polyphenols.

### 3.2. Liver Index of Mice

The results showed that there was no significant difference (*p* > 0.05) in the weight of the mice in each group (Table 2). The weight of the liver in the model group was the highest, and that in the normal group was the lowest. Silymarin and AJWTP can inhibit the liver lesions caused by CCl_4_ and increase the liver weight. Silymarin and HAJWTP can reduce the liver weight of mice with liver injury so that it is similar to that of normal mice.

### 3.3. Serum AST, ALT, TG, and TC Levels in Mice

After CCl_4_-induced liver injury, the levels of AST, ALT, TG, and TC in the serum of mice increased (model group), while the levels of AST, ALT, TG, and TC in mice with liver injury decreased after the action of silymarin and AJWTP (Table 3). Among them, the serum indexes of mice in the HAJWTP group were close to those of the silymarin and normal group mice. 

### 3.4. Serum and Hapatic MDA Content and SOD and GSH-Px Activities in Mice

The detection of the MDA content and SOD and GSH-PX activities in the serum and liver tissues of mice showed that the MDA content in the liver injury model group was the highest, while SOD and GSH-Px activities were the lowest (Table 4 and Table 5). Due to the effect of AJWTP, the MDA content decreased, SOD and GSH-Px activities increased, and the effects of the high concentration of AJWTP (HAJWTP) were stronger as compared to the low concentration of AJWTP (LAJWTP). The results indicate that the MDA content and SOD and GSH-PX activities of mice were similar to those of silymarin-treated mice and normal mice. 

### 3.5. Pathological Observation of Hepatic Tissue

**Figure 3:**
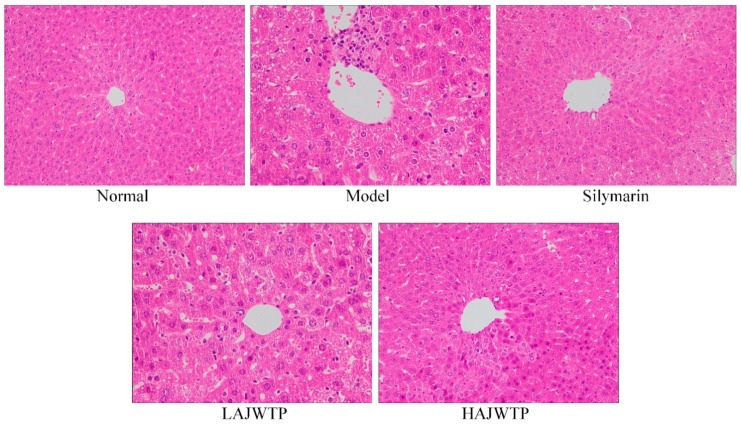
Morphological observation of hepatic tissue in mice with CCl_4_-induced liver injury. Silymarin: 200 mg/kg b.w. gavage of silymarin; LAJWTP: 100 mg/kg b.w. gavage of Anji white tea polyphenols; HAJWTP: 200 mg/kg b.w. gavage of Anji white tea polyphenols.

As shown in Figure 3, the structure of hepatic tissue cells in mice of the normal group was normal, and the central vein was independently dispersed and clearly radiated. In the mice of the model group, the structure of hepatic tissue cells was not uniform, the central vein was irregular and blurred, the structure of cells was destroyed in large quantities, and a large number of inflammatory cells had infiltrated. Both AJWTP and silymarin can alleviate the liver tissue structural damage caused by CCl_4_. The effect of high concentrations of AJWTP is obvious, and is similar to the effect observed after silymarin treatment.

### 3.6. Cyclooxygenase-2 (COX-2), Inducible Nitric Oxide Synthase (iNOS), Interleukin-1 beta (IL-1β), and Tumor Necrosis Factor alpha (TNF-α) mRNA Expression in the Hepatic Tissue of Mice

**Figure 4:**
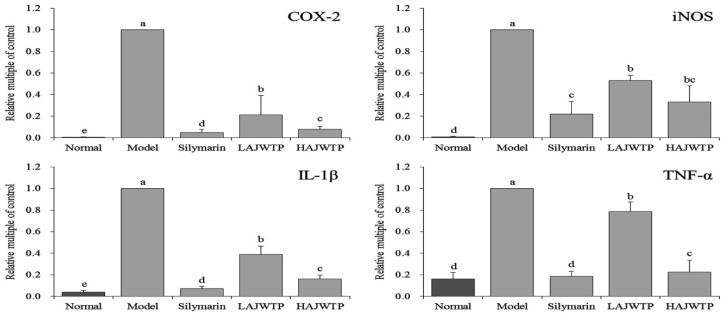
mRNA expression of cyclooxygenase-2 (*COX-2*), inducible nitric oxide synthase (*iNOS*), interleukin-1 beta (*IL-1β*), and tumor necrosis factor alpha (*TNF-α*) in the hepatic tissue of mice. Values presented are the mean ± standard deviation. ^a–e^ Mean values with different letters in the same bars are significantly different (*p* < 0.05) according to Duncan’s multiple-range test. Silymarin: 200 mg/kg b.w. gavage of silymarin; LAJWTP: 100 mg/kg b.w. gavage of Anji white tea polyphenols; HAJWTP: 200 mg/kg b.w. gavage of Anji white tea polyphenols.

The results of qPCR showed that the expression of *COX-2*, *iNOS*, *IL-1β*, and *TNF-α* was the strongest in the model group, while the expression of *COX-2*, *iNOS*, *IL-1β*, and *TNF-α* in the normal group was the weakest (Figure 4). Because AJWTP downregulated the expression of *COX-2*, *iNOS*, *IL-1β*, and *TNF-α* in mice with liver injury, and HAJWTP downregulated the additional expression of *COX-2*, *iNOS*, *IL-1β*, and *TNF-α*, the above expression in the liver tissue of mice is similar to that of silymarin-treated mice and normal mice.

### 3.7. Nuclear Factor κ-Light-Chain-Enhancer of Activated B Cells (NF-κB) and Nuclear Factor of Kappa Light Polypeptide Gene Enhancer in B-cell Inhibitor Alpha (IκB-α) mRNA Expression in the Hepatic Tissue of Mice

**Figure 5:**
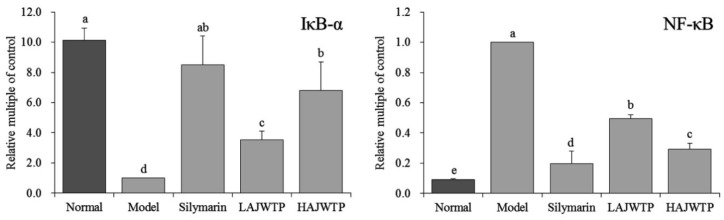
mRNA expression of *NF-κB* and *IκB-α* in the hepatic tissue of mice. Values presented are the mean ± standard deviation. ^a–e^ Mean values with different letters in the same bars are significantly different (*p* < 0.05) according to Duncan’s multiple-range test. Silymarin: 200 mg/kg b.w. gavage of silymarin; LAJWTP: 100 mg/kg b.w. gavage of Anji white tea polyphenols; HAJWTP: 200 mg/kg b.w. gavage of Anji white tea polyphenols.

The results showed that the expression of *NF-κB* was the lowest in the normal group, while the expression of *IκB-α* was the strongest in the model group (Figure 5). The expression of *NF-κB* was the strongest in the liver tissue, while the expression of *IκB-α* was the weakest in the model group. Compared with the model group, silymarin and AJWTP upregulated the expression of *IκB-α* and downregulated the expression of *NF-κB* in liver tissue, and the effect of high concentrations of AJWTP (HAJWTP) was similar to that of silymarin.

### 3.8. Cu/Zn-SOD, Mn-SOD, and CAT mRNA Expression in Hepatic Tissue of Mice

**Figure 6:**
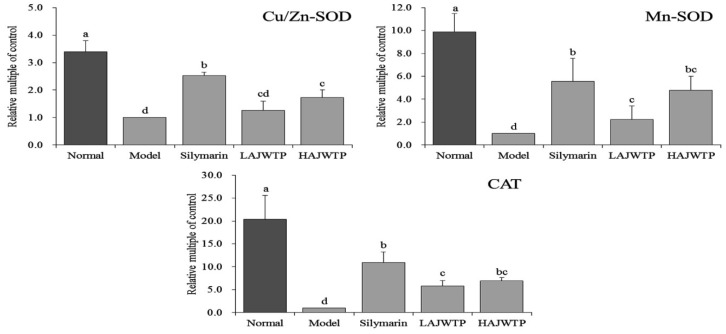
mRNA expression of copper/zinc-superoxide dismutase (*Cu/Zn-SOD*), manganese- superoxide dismutase (*Mn-SOD*), and catalase (*CAT*) in the hepatic tissue of mice. Values presented are the mean ± standard deviation. ^a–^^e^ Mean values with different letters in the same bars are significantly different (*p* < 0.05) according to Duncan’s multiple-range test. Silymarin: 200 mg/kg b.w. gavage of silymarin; LAJWTP: 100 mg/kg b.w. gavage of Anji white tea polyphenols; HAJWTP: 200 mg/kg b.w. gavage of Anji white tea polyphenols.

Figure 6 shows that the expression of *Cu/Zn-SOD*, *Mn-SOD*, and *CAT* in the liver tissue of model mice was the lowest due to the effect of CCl_4_, while that of normal mice was the strongest. The expression of *Cu/Zn-SOD*, *Mn-SOD*, and *CAT* in the liver tissue of mice in the silymarin, HAJWTP, and LAJWTP groups decreased in turn, and was stronger than that in the model group.

### 3.9. COX-2 and NF-κB Protein Expression in the Hepatic Tissue of Mice

**Figure 7:**
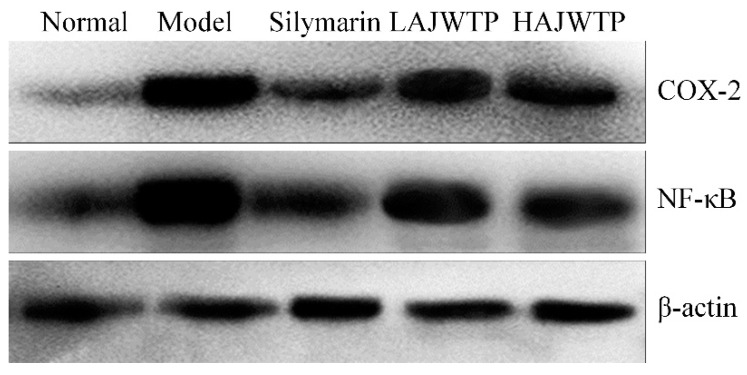
Protein expression of *COX-2* and *NF-κB* in the hepatic tissue of mice. Silymarin: 200 mg/kg b.w. gavage of silymarin; LAJWTP: 100 mg/kg b.w. gavage of Anji white tea polyphenols; HAJWTP: 200 mg/kg b.w. gavage of Anji white tea polyphenols.

The Western blot experiment results showed that the *COX-2* and *NF-κB* protein expression of mice in the model group were the strongest (Figure 7). After treatment with silymarin and AJWTP, the *COX-2* and *NF-κB* protein expression were reduced compared to the mice in the model group, and the expressions of HAJWTP treated mice were stronger than those of silymarin treated mice, but weaker than those of LAJWTP treated mice.

## 4. Discussion

The liver is a very important viscus in the body, and when it is damaged, the damage may endanger life. At present, clinical research is based on various indicators, such as the liver index, serum biochemical indicators, and liver tissue oxidation indicators, to assess the degree of liver injury. Among them, the liver index is an important indicator to measure liver injury in animal experiments, and it has been used to evaluate the degree of experimental liver injury. The changes in liver weight can directly reflect liver damage [19]. The results of this study also showed that CCl_4_ caused an increase in the organ index in mice. AJWTP effectively alleviated the increase in the liver index and resulted in the liver index of mice with liver injury being similar to that of normal mice, and the effect was also similar to that of silymarin.

Kupffer cells can produce a large amount of reactive oxygen species (ROS), and lipid peroxidation metabolites produced by CCl_4_ can also stimulate Kupffer cells, release pro-inflammatory cytokines, and further aggravate liver injury [20]. After the structure and function of the hepatocyte membrane is damaged, transaminases alanine aminotransferase (ALT) and aspartate aminotransferase (AST) overflow, which increases serum ALT and AST [21]. This reflects the degree of hepatocyte injury to a certain extent. Therefore, the elevation of serum ALT and AST is considered to be an important index for judging the severity of acute hepatic injury [22]. Liver injury can lead to the transfer of fatty acids to the liver, resulting in an increase in the TG content in the liver, while TC reacts to lipid peroxidation in the liver, and the TC level in the body increases [23]. AJWTP can reduce the levels of AST, ALT, TG, and TC caused by liver injury and alleviate liver injury caused by oxidative stress.

CCl_4_ can cause oxidative stress in the body, induce inflammation in the liver, and induce liver injury [20]. A large number of inflammatory factors, such as *TNF-α* and *IL-1β*, are released to activate the nuclear factor *NF-κB* pathway [24]. Activated NF-kappa B can promote the expression of *iNOS* and *COX-2* [25]. *iNOS* is an important inflammatory factor, and *iNOS*-induced nitric oxide (NO) also promotes further liver damage [26]. *COX-2* is an inducible enzyme that is not expressed in normal tissues. After liver injury, Kupffer cells are activated, and *COX-2* expression and synthesis occurs, which aggravates the inflammatory damage of the liver [27]. Therefore, the levels of *IL-1β* and *TNF-α* in serum can also reflect the degree of hepatocyte injury. Most of the cytoplasmic *NF-κB* in normal hepatocytes binds to *IκB-α* and is inactivated. When the body is stimulated by oxidative stress, it causes *IκB-α* phosphorylation and ubiquitination. This leads to the activation of *NF-κB*, which then enters the nucleus, mediates the transcription of the corresponding genes, and results in the occurrence of inflammation [26]. The results also showed that white tea downregulated the expression of *COX-2*, *iNOS*, *NF-κB*, *IL-1β*, and *TNF-α* in mice with liver injury and upregulated the expression of *IκB-α*, thus alleviating liver injury by inhibiting oxidative stress.

CCl_4_ induces oxidative stress in the liver tissue of mice, and CCl_4_ causes a large number of CCl_3_• free radicals in the liver. These free radicals cause acute liver injury, which severely damages hepatocytes and causes irreversible liver damage [20]. The activity of SOD in liver tissue can be used to evaluate the degree of liver injury. Regulation and promotion of SOD is the main mechanism of enzymatic antioxidant activity. Therefore, the level of SOD activity reflects the degree of liver cell injury to a certain extent [28]. SOD is a metalloenzyme that widely exists in the biological world, and it is the key line of defense for organisms against the toxicity of reactive oxygen species. According to the different metal auxiliary groups, there are three types of SOD: *Cu/Zn-SOD*, *Mn-SOD*, and Fe-SOD. *Cu/Zn-SOD* is a eukaryotic enzyme that mainly exists in the cytoplasm and chloroplast matrix of eukaryotic cells. It is found in the blood and viscera of animals. *Mn-SOD* mainly exists in the matrix of prokaryotic cells and eukaryotic cells with mitochondria, while Fe-SOD mainly exists in prokaryotic cells and a few plants [29]. SOD is effective in preventing and treating liver injury related to superoxide free radicals. When superoxide anion radicals are excessively produced or the SOD concentration is low, excessive superoxide anions will cause oxidation [30]. The levels of *Cu/Zn-SOD* and *Mn-SOD* in animals decrease when the liver is damaged by oxidation [31]. *CAT* is an antioxidant enzyme that mainly exists in erythrocytes and some tissue cells, and also in mitochondria and the cytoplasm [32].

In the process of normal oxidative respiration, organisms constantly produce ROS. As a highly active molecule, it contains unpaired electrons, and thus, *CAT*, SOD, and GSH-Px and other enzymatic systems remove ROS. SOD is the first line of defense to eliminate ROS, and it mainly disproportionates O_2_^−^ to H_2_O_2_. *CAT* can decompose H_2_O_2_ to produce H_2_O, and increase the oxygen content in cells [33]. GSH-Px is one of the most important antioxidants in mammals, and it can scavenge free radicals produced in cells, thereby reducing the damage to the cell membrane caused by the formation of reactive oxygen species through lipid peroxidation [34]. GSH-Px is directly or indirectly involved in many life activities of microbial cells. One of the most important roles of GSH-Px is to co-inhibit the oxidative stress response of cells and slow the oxidation of organisms with related metabolic enzymes [35]. MDA is the end product of lipid oxidation. It is one of the most important products of membrane lipid peroxidation. The degree of membrane lipid peroxidation can be judged by MDA, and the degree of oxidative damage can be indirectly measured. MDA is also a sensitive index of liver injury [36]. In this study, AJWTP prevented liver injury by reducing CCl_4_-induced oxidative stress, enhancing SOD, *CAT*, and GSH-Px activity, and inhibiting MDA.

## 5. Conclusions

This study proved that AJWTP exhibited a strong preventive effect on CCl_4_-induced liver injury in mice. Compared with the case without AJWTP treatment for prevention, the liver injury of model mice was more serious. It was found that AJWTP effectively controlled the level of oxidative stress in mice by detecting the biochemical indicators in serum and liver tissues and the expression of mRNA and protein in liver tissues, and prevented liver damage caused by oxidative stress from CCl_4_. AJWTP has a strong inhibitory effect of oxidative stress and a preventive effect on liver injury induced by CCl_4_, and its effect is similar to that of silymarin. It can function as an active substance with antioxidant and liver protection potential.

## Figures and Tables

**Table 1:** Sequences of primers used in the quantitative polymerase chain reaction (qPCR) assay.

Gene Name	Sequence
*COX-2*	Forward: 5′-GGTGCCTGGTCTGATGATG-3′
	Reverse: 5′-TGCTGGTTTGGAATAGTTGCT-3′
*iNOS*	Forward: 5′-GTTCTCAGCCCAACAATACAAGA-3′
	Reverse: 5′-GTGGACGGGTCGATGTCAC-3′
*IL-1β*	Forward: 5′-CTCCATGAGCTTTGTACAAGG-3′
	Reverse: 5′-TGCTGATGTACCAGTTGGGG-3′
*TNF-α*	Forward: 5′-GACCCTCAGACTCAGATCATCCTTCT-3′
	Reverse: 5′-ACGCTGGCTCAGCCACTC-3′
*NF-κB*	Forward: 5′-ATGGCAGACGATGATCCCTAC-3′
	Reverse: 5′ CGGAATCGAAATCCCCTCTGTT-3′
*IκB-α*	Forward: 5′-TGAAGGACGAGGAGTACGAGC-3′
	Reverse: 5′-TGCAGGAACGAGTCTCCGT-3′
*Cu/Zn-SOD*	Forward: 5′-AACCAGTTGTGTTGTCAGGAC-3′
	Reverse: 5′-CCACCATGTTTCTTAGAGTGAGG-3′
*Mn-SOD*	Forward: 5′-CAGACCTGCCTTACGACTATGG-3′
	Reverse: 5′-CTCGGTGGCGTTGAGATTGTT-3′
*CAT*	Forward: 5′-GGAGGCGGGAACCCAATAG-3′
	Reverse: 5′-GTGTGCCATCTCGTCAGTGAA-3′
*GAPDH*	Forward: 5′-AGGTCGGTGTGAACGGATTTG-3′
	Reverse: 5′-GGGGTCGTTGATGGCAACA-3′

**Table 2:** Effects of Anji white tea polyphenols (AJWTP) on the body weight, liver weight, and liver index of mice with hepatic injury induced by CCl_4_ (*N* = 10).

Group	Body Weight (g)	Liver Weight (g)	Liver Index
Normal	38.776 ± 5.485 ^a^	1.422 ± 0.107 ^c^	3.651 ± 0.361 ^e^
Model	39.351 ± 2.036 ^a^	2.278 ± 0.158 ^a^	5.618 ± 0.339 ^a^
Silymarin	39.257 ± 2.890 ^a^	1.587 ± 0.155 ^b^	4.046 ± 0.045 ^d^
LAJWTP	38.223 ± 2.557 ^a^	2.011 ± 0.127 ^a^	5.082 ± 0.092 ^b^
HAJWTP	37.432 ± 3.994 ^a^	1.578 ± 0.085 ^b^	4.459 ± 0.033 ^c^

Values presented are the mean ± standard deviation. ^a–^^e^ Mean values with different letters in the same row are significantly different (*p* < 0.05) according to Duncan’s multiple-range test. Silymarin: 200 mg/kg b.w. gavage of silymarin; LAJWTP: 100 mg/kg b.w. gavage of Anji white tea polyphenols; HAJWTP: 200 mg/kg b.w. gavage of Anji white tea polyphenols.

**Table 3:** The levels of aspartate aminotransferase (AST), alanine aminotransferase (ALT), triglyceride (TG), and total cholesterol (TC) in the serum of mice with hepatic injury induced by CCl_4_ (*N* = 10).

Group	AST (Karmen/mL)	ALT (Karmen/mL)	TC (mg/dL)	TG (µmol/dL)
Normal	29.49 ± 3.09 ^e^	18.30 ± 0.84 ^e^	101.67 ± 28.43 ^b^	58.23 ± 2.40 ^b^
Model	303.65 ± 15.65 ^a^	361.55 ± 1.69 ^a^	165.00 ± 47.7 ^a^	67.71 ± 10.65 ^a^
Silymarin	155.30 ± 6.06 ^d^	42.74 ± 15.08 ^d^	105.00 ± 25.98 ^b^	63.99 ± 3.14 ^ab^
LAJWTP	256.75 ± 4.28 ^b^	294.49 ± 0.82 ^b^	130.00 ± 20.00 ^ab^	66.37 ± 0.68 ^a^
HAJWTP	170.48 ± 5.28 ^c^	112.60 ± 33.11 ^c^	113.33 ± 30.14 ^b^	64.29 ± 1.61 ^ab^

Values presented are the mean ± standard deviation. ^a–^^e^ Mean values with different letters in the same row are significantly different (*p* < 0.05) according to Duncan’s multiple-range test. Silymarin: 200 mg/kg b.w. gavage of silymarin; LAJWTP: 100 mg/kg b.w. gavage of Anji white tea polyphenols; HAJWTP: 200 mg/kg b.w. gavage of Anji white tea polyphenols.

**Table 4:** The content of malondialdehyde (MDA) and superoxide dismutase (SOD) and glutathione peroxidase (GSH-Px) activities in the serum of mice with hepatic injury induced by CCl_4_ (*N* = 10).

Group	SOD (U/mL)	GSH-Px (U/mL)	MDA (nmol/mL)
Normal	218.71 ± 10.66 ^a^	221.85 ± 12.08 ^a^	3.39 ± 0.41 ^e^
Model	59.72 ± 4.32 ^e^	70.89 ± 5.25 ^e^	37.65 ± 1.71 ^a^
Silymarin	148.38 ± 7.59 ^b^	155.29 ± 7.16 ^b^	13.57 ± 0.89 ^d^
LAJWTP	79.81 ± 5.50 ^d^	82.55 ± 5.73 ^d^	28.99 ± 1.62 ^b^
HAJWTP	122.36 ± 6.63 ^c^	138.71 ± 4.39 ^c^	18.37 ± 1.30 ^c^

Values presented are the mean ± standard deviation. ^a–^^e^ Mean values with different letters in the same row are significantly different (*p* < 0.05) according to Duncan’s multiple-range test. Silymarin: 200 mg/kg b.w. gavage of silymarin; LAJWTP: 100 mg/kg b.w. gavage of Anji white tea polyphenols; HAJWTP: 200 mg/kg b.w. gavage of Anji white tea polyphenols.

**Table 5:** The content of MDA and SOD and GSH-Px activities in the hepatic tissue of mice with hepatic injury induced by CCl_4_ (*N* = 10).

Group	SOD (U/mg prot)	GSH-Px (U/mg prot)	MDA (nmol/mg prot)
Normal	93.57 ± 5.87 ^a^	192.67 ± 8.71 ^a^	1.03 ± 0.12 ^e^
Model	21.32 ± 3.88 ^e^	48.36 ± 4.30 ^e^	9.15 ± 0.43 ^a^
Silymarin	79.35 ± 4.79 ^b^	125.77 ± 6.68 ^b^	3.41 ± 0.25 ^d^
LAJWTP	40.98 ± 3.87 ^d^	73.58 ± 5.29 ^d^	7.05 ± 0.28 ^b^
HAJWTP	66.72 ± 5.05 ^c^	112.33 ± 4.21 ^c^	4.32 ± 0.21 ^c^

Values presented are the mean ± standard deviation. ^a–^^e^ Mean values with different letters in the same row are significantly different (*p* < 0.05) according to Duncan’s multiple-range test. Silymarin: 200 mg/kg b.w. gavage of silymarin; LAJWTP: 100 mg/kg b.w. gavage of Anji white tea polyphenols; HAJWTP: 200 mg/kg b.w. gavage of Anji white tea polyphenols.
